# Microalgae and cyanobacteria as a tool for agricultural sustainability: a review of biofertilizer and biostimulant potential

**DOI:** 10.3389/fpls.2025.1733394

**Published:** 2026-01-12

**Authors:** Zhongliang Sun, Xiaoyan Liu, Adamu Yunusa Ugya, Haiyan Liu, Liqin Sun, Guanghong Luo

**Affiliations:** 1 School of Life Sciences, Yantai University, Yantai, China; 2 Gansu Microalgae Technology Innovation Center, Key Laboratory of Hexi Corridor Resources Utilization of Gansu, Hexi University, Zhangye, China; 3 Gansu Kaiyuan Biotechnology Development Center Co., Ltd., Zhangye, China; 4 School of Life Sciences, Henan University, Kaifeng, China

**Keywords:** biopesticide, circular economy, economic viability, phytohormones, sustainable agriculture

## Abstract

Microalgae and cyanobacteria are emerging as sustainable alternatives to chemical fertilizers and pesticides, offering nutrient recycling, stress mitigation, and environmental restoration within the framework of circular bioeconomy. This review synthesizes recent advances in the utilization of cyanobacteria and green microalgae as biofertilizers, biostimulants, and biopesticides, emphasizing their physiological mechanisms and agronomic potential. Microalgae and cyanobacteria can fix atmospheric nitrogen, solubilize phosphorus, and supply essential micronutrients through exopolysaccharides, organic acids, and siderophores, thereby improving soil fertility and structure. Their metabolites, including phytohormones, amino acids, and antioxidants, stimulate seed germination, root growth, nutrient uptake, and tolerance to abiotic stresses such as drought and salinity. Moreover, allelochemicals and antimicrobial compounds from microalgae can suppress plant pathogens and reduce pesticide dependence. Integrating microalgae cultivation with wastewater and flue gas utilization promotes nutrient recycling and CO_2_ sequestration, further enhancing environmental sustainability. However, large-scale application remains limited by biomass production costs, inconsistent performance under field conditions, and regulatory uncertainty. Overall, microalgae-based fertilizers and biostimulants hold great promise for sustainable crop production and soil health improvement. Future research should focus on low-cost cultivation and harvesting technologies, field scale validation, and standardized product formulations to accelerate the transition toward climate smart and resource sustainable agriculture.

## 1 Introduction

The exponential increase in the human population has placed immense pressure on the Earth’s resources and ecosystems (Tal, 2025). Particularly food scarcity and water shortages, which have become more prevalent in many regions around the world (Saleem et al., 2025). Food scarcity results from the decline in natural soil fertility due to the insufficiency of space for other traditional systems of farming, such as shifting cultivation and slash-and-burn agriculture (Serrani et al., 2022). Also, population blooms have led to increased competition for limited land resources for other purposes such as urbanization and industrialization, further exacerbating the issue of food scarcity. This has led to an increased reliance on intensive farming practices such as monoculture and the use of chemical fertilizers and pesticides to maximize crop yields (Satterthwaite et al., 2010). This is because chemical fertilizers such as nitrogen, phosphorus, and potassium provide a direct, potent, and rapidly available nutrient source that boosts crop productivity, ensuring that more food can be grown on existing farmland (Govindasamy et al., 2023). The nitrogen provided by fertilizers promotes vigorous leaf and stem growth, resulting in a larger, more productive plant. Adequate phosphorus is essential for root development and overall plant health, while potassium helps regulate water uptake and nutrient transport within the plant. Therefore, fertilizer helps to correct soil deficiencies and imbalances, ensuring that crops have access to all the necessary nutrients for optimal growth. This increases yield per unit of land and contributes to global food security by maximizing the efficiency of agricultural production (Ma et al., 2023).

Continuous dependence on chemical products for agricultural practices has created an inconsistency that boosts short-term yield and creates long-term ecological consequences (Ahmad et al., 2024). For example, the Haber-Bosch process for ammonia synthesis consumes vast amounts of fossil fuels, contributing to greenhouse gas emissions and climate change. Also, only about 30-50% of applied N-P-K fertilizers are actually taken up by crops, with the rest leaching into waterways or remaining in the soil, leading to nutrient pollution and degradation of ecosystems (Laje et al., 2019). This highlights the need for the growing interest in sustainable farming practices such as crop rotation and organic farming. Despite its substantial role in enhancing soil health and decreasing dependence on environmentally detrimental agrochemicals (Al-Shammary et al., 2024). The transition to sustainable farming practices can be difficult for farmers due to initial costs and potential decrease in yields during the transition period (Gamage et al., 2024). The switch towards biofertilizers and biostimulants is a better and easy alternative for farmers looking to adopt sustainable practices without sacrificing productivity. This is because the transition to biofertilizers and biostimulants can help improve soil health and crop yield while also reducing the reliance on synthetic fertilizers and pesticides (Miranda et al., 2024). The gradual incorporation of these products into farming practices enhance the benefits of sustainable agriculture without causing major financial or yield challenges (Roy and Medhekar, 2025). This is because biofertilizers and biostimulants allow the continuous cultivation of crops of interest without the need for crop rotation, leading to increased efficiency and cost savings in the long run (Mahmud et al., 2021). Despite the significance of biostimulants and biofertilizers, issues such as variable environmental factors, poor adaptability, and competition with native microbes hinder the use of biofertilizers and biostimulants in agricultural industries (Ferreyra-Suarez et al., 2024). To counteract this limitation, it is crucial to focus on improving the formulation and effectiveness of biofertilizers and biostimulants using different biomass. The identification of specific strains of microbes that are better suited for different environmental conditions will help enhance the performance and efficacy of biofertilizers and biostimulants in agricultural settings (Boutahiri et al., 2024).

Different bioresources have been used as biofertilizers and biostimulants to enhance crop productivity and soil health. For example, Plant Growth-Promoting Rhizobacteria (PGPR) have been shown to fix atmospheric nitrogen, solubilize phosphorus/potassium, produce phytostimulants, and improve plant growth and stress tolerance (Khoso et al., 2024). Humic substances have been linked to the enhancement of soil structure by increasing nutrient and water retention, leading to the stimulation of root growth and plant metabolic processes (Trevisan et al., 2010). Seaweed extracts have been shown to be rich in micronutrients, amino acids, and plant hormones that can enhance plant growth, yield, and stress tolerance (Yasmeen et al., 2025). Whereas protein hydrolysates have been shown to provide nitrogen in a readily absorbable form, which can promote rapid plant growth and improve overall plant health (Colla et al., 2017). Microalgae and cyanobacteria are a superior option for use in biofertilizers and biostimulants due to their unique properties that enable the dual function of simultaneously acting as both a biofertilizer and a biostimulant (Nur et al., 2025). This is because they have the ability to provide essential nutrients and also the capability to produce a range of bioactive compounds, such as phytohormones, that are linked to the enhancement of plant growth, development, and stress resilience (Ugya et al., 2024a). Also, the potential circularity of microalgae cultivation makes them a sustainable and environmentally friendly option for agricultural practices, as they can be grown using waste nutrients and carbon dioxide from various industries (Ugya et al., 2024c, 2023). This not only reduces the environmental impact but also contributes to the overall sustainability of food production systems (Rahman et al., 2025). Despite the significance of microalgae resources as a tool for biofertilizers and biostimulants, challenges such as scale-up production, cost viability, harvesting, and processing have limited the widespread adoption of these sustainable alternatives in agriculture (Samoraj et al., 2024). This review aimed to identify the key strategies and technologies that can address these challenges and promote the successful integration of microalgae-based biofertilizers and biostimulants in agricultural practices.

## 2 Mechanism of microalgae and cyanobacteria resources as a biofertilizer

Microalgae and cyanobacteria have gained significant attention as sustainable biofertilizers due to their rich nutrient content, growth-promoting properties, and environmental benefits (Gonçalves et al., 2023). The presence of proteins, amino acids, lipids, polysaccharides, vitamins, and minerals makes them ideal for use in agriculture. Microalgae and cyanobacteria are able to supply macro- and micronutrients such as nitrogen, phosphorus, potassium, iron, and magnesium, which are essential for plant growth and development (Ugya et al., 2024b). Also, microalgae and cyanobacteria can help improve soil structure and enhance water retention, leading to increased crop yields and overall soil health. Their ability to produce antimicrobial compounds that suppress soil-borne pathogens also contributes to healthier plants and reduced need for chemical pesticides (Ramakrishnan et al., 2023).

**Figure 1:**
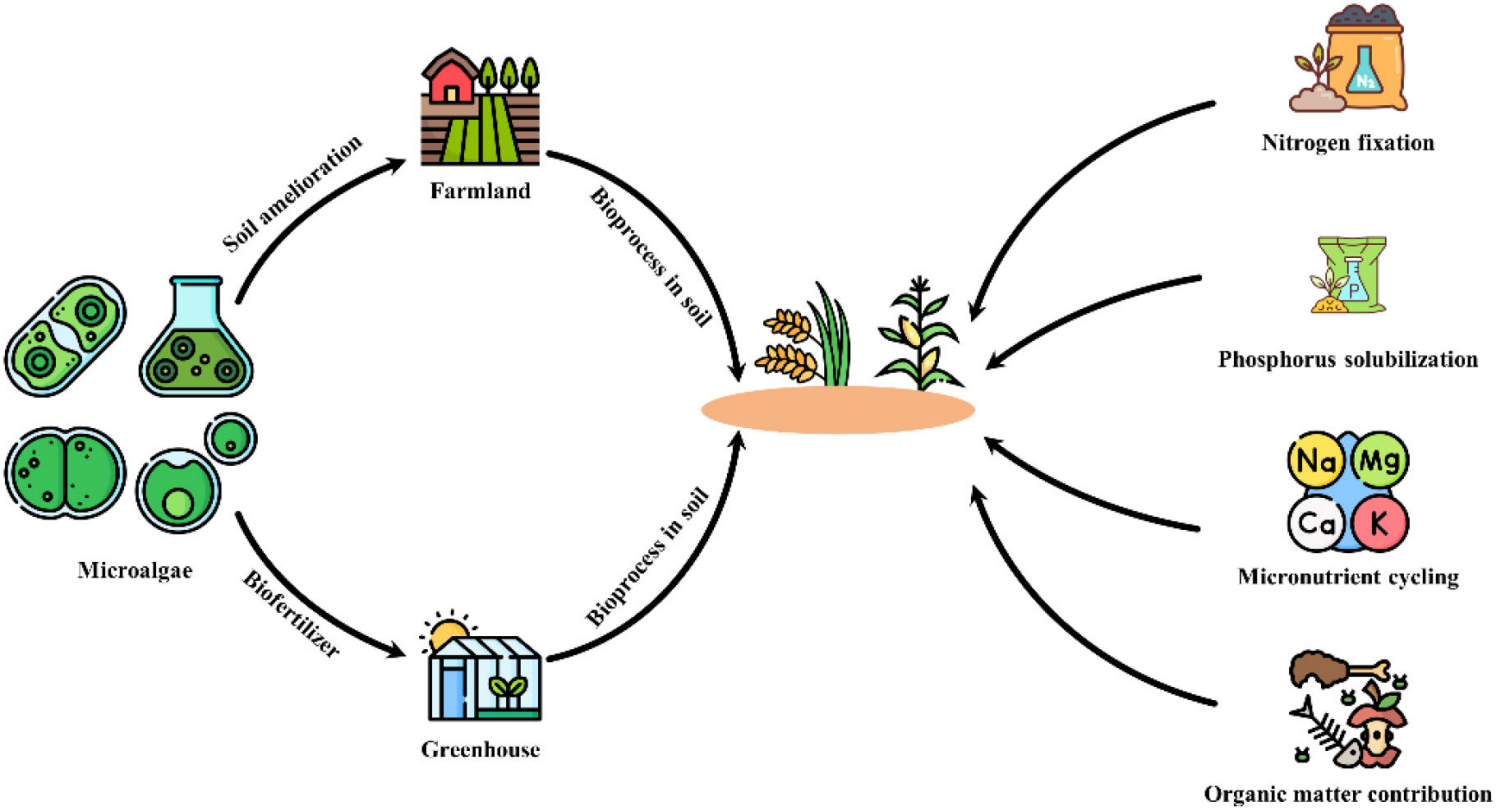
Microalgae-to-farmland circular route and the four mechanisms by which microalgal biofertilizers promote plant performance. The schematic depicts a circular pathway from microalgae cultivation (left) to biofertilizer production and application, followed by in-soil bioprocesses that enhance crop growth (center). Right-hand arrows summarize four core mechanisms: (i) nitrogen fixation by cyanobacteria and diazotrophic consortia; (ii) phosphorus solubilization driven by CO_2_ uptake–induced pH shifts, organic acids, and extracellular polymeric substances (EPS) and their interactions with phosphate-solubilizing microbes; (iii) micronutrient cycling through siderophores/EPS that mobilize Fe, Zn, Mn, Cu and other elements; and (iv) organic matter contribution via algal biomass and exudates that improve soil aggregation, water retention, and microbial activity. Together, these processes couple soil amelioration with crop productivity, aligning microalgal inputs with a circular bioeconomy framework.

The benefit of using microalgae and cyanobacteria for agriculture is not only their nutrient-rich composition but also their potential to contribute to sustainable farming practices by reducing the reliance on synthetic fertilizers and pesticides. Additionally, microalgae cultivation can be done in a way that minimizes environmental impact, making them a promising option for eco-friendly agriculture. Whereas the production of synthetic fertilizer and pesticides leads to negative carbon footprints (Jurado-Flores et al., 2025). Different studies have reported the positive impact of microalgae on crop yield and quality, as well as their ability to enhance soil health and biodiversity. By incorporating microalgae into agricultural practices, farmers can not only improve the health of their crops but also contribute to a more sustainable and environmentally friendly food production system. For example, Zhang et al. reported that the use of extracts from *Chlorella* sp. and *Anabaena* sp. causes 81.7% and 58.3% increases in the length of cucumber seedling stems, respectively. The microalgal extract inhibited primary root elongation while concurrently promoting the development of lateral and fibrous root systems (Zhang et al., 2024b). The ideal of microalgae biofertilizer is to provide essential nutrients to crops through direct and indirect mechanisms. This mechanism includes nitrogen fixation, phosphorus solubilization, potassium and micronutrient cycling, and organic matter contribution as shown in **Figure 1**.

### 2.1 Nitrogen fixation–driven biofertilization by microalgae and cyanobacteria

Microalgae are a critical component of the global nitrogen cycle through the conversion of atmospheric nitrogen into a usable form for plants and other organisms (Alvarez et al., 2021). For example, cyanobacteria are known for their ability to fix nitrogen, making it available for other organisms in the ecosystem. This process is essential for maintaining healthy soil and promoting plant growth in various environments (Nawaz et al., 2024a). Filamentous cyanobacteria such as *Trichodesmium* sp.*, Anabaena* sp., and *Nostoc* sp. have specialized heterocysts that allow them to carry out nitrogen fixation efficiently (Parab and Matondkar, 2012). These heterocysts create a low-oxygen environment that is essential for the enzyme nitrogenase to function properly. This enzyme is responsible for converting atmospheric nitrogen gas (N_2_) into ammonia (NH_3_) through an energy-intensive reaction (Nawaz et al., 2024b). Heterocysts are able to create an anaerobic environment for the nitrogenase enzyme due to their thickened cell wall layers. This thick cell wall is made up of glycolipid and polysaccharide layers, which act as a diffusion barrier to oxygen, thus reducing the rate at which it enters the cell. The inactivation of photosystem II in heterocysts also helps to maintain the anaerobic conditions necessary for nitrogen fixation. This allows for efficient conversion of atmospheric nitrogen into ammonia by the nitrogenase enzyme (Black and Osborne, 2004). Other non-filamentous cyanobacteria, such as *Crocosphaera* sp. and *Cyanothece* sp., are able to fix nitrogen through the temporal separation of photosynthesis and nitrogen fixation processes. By performing these functions at different times of day or night, these cyanobacteria can optimize their use of resources and energy to support growth and survival in nutrient-poor environments. This adaptation allows them to thrive in diverse ecosystems, contributing significantly to the global nitrogen cycle (Umbricht et al., 2024). The application of microalgae in nitrogen fixing for enhanced agricultural productivity has shown promising results, as shown in **Table 1**. The utilization of the unique ability of microalgae to convert atmospheric nitrogen into a form usable by plants can potentially reduce their reliance on synthetic fertilizers and improve soil health in a sustainable manner. These microalgae can be inoculated to the soil in the form of dried or liquid cultures added to soil before or during planting. They can also be inoculated in water, as is common in rice fields for *Azolla–Anabaena* systems. Some seeds are coated with algal biofertilizer to provide a slow-release source of nitrogen throughout the growing season. This innovative approach has the potential to revolutionize agricultural practices by promoting eco-friendly and cost-effective solutions for farmers worldwide. For example, the use of *Azolla*-*Anabaena* symbiosis in rice cultivation has been shown to increase rice yields while also reducing the environmental impact of nitrogen runoff. This symbiotic relationship between the two organisms provides a natural and eco-friendly alternative to traditional fertilization methods (Kannaiyan and Ganesan, 2002).

**Table 1:** Effectivity of microalgae in nitrogen fixation.

Microalgae species	Application conditions	Effect on nitrogen fixing/plant growth	Reference:
*Anabaena cylindrica* *Chlamydomonas* sp.	Spring wheat growth	The treatment of soil with a higher inoculum of 100% nitrogen from *Anabaena cylindrica* resulted in a significant increase in nitrate/nitrite levels of the surface soil.	(Alvarez et al., 2024)
*Westiellopsis prolifica* *Anabaena variabilis*	Both microalgae were exposed to insoluble tricalcium phosphate and Mussorie rock phosphate	*Anabaena* variabilis best solubilized insoluble tricalcium phosphate of 20 mg P/L, leading to higher phosphorus solubilization and nitrogen fixing rate.	(Yandigeri et al., 2011a)
*Nostoc* sp.	*Noctoc* sp. suspension was applied in potted soil at 6 g/dry weight per square meter	The nitrogen in the soil increase by by 17% and 40% in Hertzog and Guquka soils, respectively	(Maqubela et al., 2009)
*Anabaena azotica Tolypothrix tenuis*	Use in a potting experiment to monitor rice growth in saline environments.	The microalgae fixed nitrogen, improving plant height, and leaf total chlorophyll content by 4.42% and 19.46%.	(Zhang et al., 2025c)
*Anabaena azotica*	Microalgae biomass was used to enhance the growth of of rice and soil carbon sequestration.	Rice yield reach 38–74% in high-yielding soils and 107–157% in low-yielding soils. Available nitrogen content, *N*-acetyl-β-glucosaminidase, activities of soil acid phosphatase and soil pH also increase.	(Li et al., 2025)
*Nostoc* sp	The efficacy of *Nostoc* sp. in association with *Delftia lacustris* in nitrogen fixation in rice cultivation was assessed in greenhouse experiments.	The total nitrogen, ammonium, phosphorus and potassium was increased by 37.8%, 42.1%, 35.01%, and 15.36%, respectively, compared to controls.	(Gholami et al., 2025)
*Nostoc* sp	Wheat growth in artificial semi-arid soil	Nostoc biomass improved the water holding capacity of the soil, allowing wheat plants to better withstand drought conditions	(Do Nascimento et al., 2019)
*Nostoc kihlmani Anabaena cylindrica*	The role of both microalgae in improvig the properties of degraded soil was investigated.	The treatment of the soil using the microalgae led to an improvement in the level of nitrogen, phosphorus, potassium, and total organic carbon of the soil.	(Gheda and Ahmed, 2015)
*Anabaena cylindrica* *Nostoc calcicola*, *Nostoc muscorum*	The growth-promoting effect of the microalgae on wheat and *Arabidopsis thaliana* was investigated in both hydroponic and pot culture experiments.	*Nostoc calcicola enhanced the* higher dry mass of wheat plants by more than 30% and 44% in hydroponics and pot culture conditions. Whereas under drought stress, *Nostoc muscorum* and *Nostoc calcicola* improved the dry weight of *Arabidopsis thaliana* by 57% and 32%, respectively.	(Kollmen et al., 2025)
*Chlorella vulgaris*	The efficacy of *Chlorella vulgaris* in enhancing the growth of wheat was conducted in a greenhouse experiment.	The microalgae was used to remove 43.65% and 75.42% of nitrogen and phosphorus from wastewater, and the 0.04 g kg^-1^ biomass produced was combined with *Diammonium* Phosphate fertilizer for the greenhouse cultivation of wheat. The result showed significant improvement in soil properties and plant growth parameters.	(Akca et al., 2025)

### 2.2 Phosphorus solubilization and mobilization by microalgae and cyanobacteria

Although microalgae and cyanobacteria cannot directly trigger the solubilization of insoluble phosphorus compared to certain bacteria and fungi. They produce large amounts of organic acids that create environmental conditions that lead to the dissolution of insoluble phosphorus as shown in **Table 2**. This is because the organic compounds released by microalgae can chelate with phosphorus, making it more bioavailable for plants (Ugya et al., 2025c). The key mechanisms of phosphorus solubilization by microalgae include carbon dioxide (CO_2_) assimilation and pH increase, organic acid production, production of exopolymeric substances (EPS), and symbiotic relationships with phosphate-solubilizing microorganisms (PSM). Microalgae and cyanobacteria are able to capture CO_2_ from their environment, leading to a shift towards a more alkaline pH that enhances the solubilization of phosphorus. This is because in alkaline conditions, insoluble calcium-bound phosphates such as Ca_3_(PO_4_)_2_ and hydroxyapatite become more soluble, leading to the release of phosphate ions (PO_4_^3-^) into the environment, making it available for uptake. This process can be effectively stimulated in calcareous soils and sediments to enhance agricultural productivity and improve nutrient availability for plants (Marks et al., 2019). The key process in which microalgae and cyanobacteria are able to trigger phosphorus solubilization is through a symbiotic interaction with PSM. In this relationship they provide PMS with organic carbon in the form of exudates and oxygen from photosynthesis to fuel their metabolic activity. In return, PSM release enzymes that help break down organic phosphorus compounds in the soil, making phosphorus more available for uptake by the microalgae. This mutualistic relationship has been used to enhance nutrient cycling and agricultural productivity in various ecosystems, demonstrating the potential for sustainable and eco-friendly agricultural practices (Solovchenko et al., 2021). During this symbiotic relationship, microalgae and other microorganisms produce EPS in the form of a slimy matrix of exopolymers, primarily composed of polysaccharides and proteins. EPS plays a crucial role in soil aggregation, water retention, and nutrient cycling, benefiting plant growth and overall soil health. Additionally, EPS can also help sequester carbon in the soil, contributing to climate change mitigation efforts (Costa et al., 2018).

**Table 2:** Mechanism of microalgae in phosphorus solubilization.

Microalgae species	Organic phosphorus source	Effect on phosphorus solubilization	Reference
*Anabaena* sp.	Bone meal and rock phosphate	The phosphorus solubilization potential of *Anabaena* sp. was more effective than the Mammoth P bacterial consortium.	(Afkairin et al., 2021)
*Anabaena cylindrica* *Chlamydomonas* sp.	–	Higher inoculum rates of *Chlamydomonas* sp. enhance available phosphorus levels.	(Alvarez et al., 2024)
*Westiellopsis* sp. *Anabaena* sp.	Substrate was Mussorie rock phosphate and tricalcium phosphate	Indicates the use of phthalic acid as a possible mechanism of phosphorus solubilization.	(Yandigeri et al., 2011b)
*Nostoc* sp. *Calothrix* sp. *Trichormus* sp.	–	7.1% of the tested strains of Nostoc sp. were shown to have a high phosphate solubilizing potential.	(Toribio et al., 2020)
*Anabaena variabili* *Nostoc muscorum* *Tolypothrix tenuis* *Aulosira fertilissima* *Westiellopsis prolifica*	Insoluble tricalcium phosphate and Mussorie rock phosphate	Comparation of the phosphorus solubilization of the microalgae show that *Anabaena variabili* have the highest phosphorus solubilization rate with available phosphorus of 0.224 µg/ml.	(Mishra et al., 2019)

The ability of microalgae and cyanobacteria to trigger phosphorus solubilization increases its suitability for use as a biofertilizer to improve soil health. For example, Su et al. (2025) reported that the use of *Chlorella pyrenoidosa* and *Tolypothrix tenuis* as a biofertilizer during the cultivation of *Polygala tenuifolia* resulted in the high yield. The increase in the yield of *Polygala tenuifolia* was due to the fact that the microalgae trigger a 15.68% increase in soil organic matter content. The use of microalgae in place of chemical fertilizer also causes an increase in the relative abundance of *Chloroflexi* sp. while decreasing the relative abundance of *Cladosporium* sp (Su et al., 2025). Microalgae are able to increase soil fertility by improving soil phosphorus availability through raising soil pH to solubilize Ca-P complexes. This process allows for better absorption of phosphorus by plants, leading to improved growth and productivity. Also, microalgae also contribute to soil organic matter accumulation, leading to the release of EPS that act as binding agents, promoting synergistic interactions between soil particles and microorganisms. Increasing the availability of phosphorus, particularly from calcium-phosphate (Ca-P) complexes, creates a more favorable chemical environment for nutrient uptake by plants. Whereas, the release of EPS causes the improvement of soil structure and water retention. The EPS also acts as a carbon source that stimulates synergistic microbial activity, which enhances physical soil properties and promotes a healthy, functional soil microbiome (Solovchenko et al., 2019). Also, the ability of microalgae to store phosphorus as intracellular polyphosphates also contributes to its biofertilizer potential, as these polyphosphates can be released back into the soil upon cell death, providing a slow-release source of phosphorus for plant uptake. This sustainable nutrient cycling helps to improve soil fertility and overall plant health in agricultural systems (Bossa et al., 2024). Microalgae can also be utilized for the dual function of phosphorus removal from wastewater and use as biofertilizer. For example, Luo et al. (2025) reported that the 16-day cultivation of *Chlorella* sp. in high-fluoride-phosphoric wastewater led to the decrease in phosphorus from 12.76 mg/L to 5.00 mg/L. This decrease in phosphorus was attributed to the ability of the microalgae to uptake the phosphorus into the cell or absorb it to its functional group on the EPS. Once harvested, this nutrient-rich microalgae can be applied directly to soil as a biofertilizer, providing essential nutrients for plant growth. Also, the use of microalgae for wastewater treatment can help reduce environmental pollution and promote sustainable agricultural practices (Luo et al., 2025).

### 2.3 Enhancement of soil micronutrients and organic matter through the application of microalgae and cyanobacteria as biofertilizer

The application of microalgae and cyanobacteria to soil, either as living cultures, biomass extracts, or compost additives, provides micronutrients, organic matter, and bioactive compounds that enhance plant growth and improve soil health. For example, the application of *Chlorella* sp. and *Scenedesmus* sp. has been shown to significantly increase the level of Iron which is useful for improving chlorophyll production in plants (Barahoei et al., 2024). Whereas *Limnospira platensis* has been shown to produce zinc, which is important in enzyme activation and auxin synthesis (Shedeed et al., 2022). Other microelement supplies to soil by microalgae include manganese, copper, boron, and molybdenum. These essential elements enhanced soil fertility and plant nutrition, leading to increased crop yields and overall sustainability in agricultural practices (Jiménez-Ríos et al., 2024). Microalgae are able to maintain the presence of these essential elements in the soil through the secretion of organic compounds such as siderophores and EPS (Umamaheswari et al., 1997; Moreno et al., 1998). These organic compounds then chelate or bind essential metal nutrients leading to the transformation of metal nutrients into bioavailable forms that can be utilized by microalgae, plants, and other soil organisms (Ghorbani et al., 2022). The first step of this process involves the sensing of essential metal nutrient deficiency by microalgae. This then causes the activation of genes responsible for the production of siderophores and EPS. The siderophores diffuse towards iron oxide particles. They bind to the iron with an affinity so high that they can dissolve the mineral. The resulting Fe^3+^-siderophore complex is highly stable and soluble in water. This allows for easier uptake of iron by microalgae and other organisms in the soil, promoting growth and productivity. While the EPS acts as a cation exchange resin, trapping and concentrating metal ions from the soil solution and directly from mineral surfaces. This process is less specific than siderophores but highly effective at creating a localized reservoir of bioavailable nutrients. The outer membrane receptors in microalgae aid in the recognition of the Fe^3+^-siderophore complex, while periplasmic binding protein facilitates the transport of iron into the cell. ATP-binding cassette transporter then translocates the iron across the inner membrane and delivers it to the cytoplasm, where it can be utilized for various cellular processes. This process ensures that microalgae have a sufficient supply of iron for growth and metabolism (Giner-Lamia et al., 2016). Metal nutrients bound to EPS are directly absorbed or processed by enzymes associated with the biofilm matrix, allowing microalgae to efficiently uptake these essential metal nutrients from their environment (Faruque et al., 2024). When microalgae is used as biofertilizer, these nutrients can help improve soil fertility and directly or indirectly promote plant growth. Plants can directly take up Fe^3+^-siderophore complexes or indirectly through the release of organic acids by microalgae that solubilize iron in the soil, making it more available for plant uptake (Song et al., 2024).

The use of microalgae and cyanobacteria biomass as biofertilizer also increases the organic carbon and biopolymer content of the soil. These organic components trigger an increase in microbial activity, leading to enhanced metal nutrient mineralization and improved soil structure (De Silva et al., 2024). The pathways through which the use of microalgae biomass enhances soil organic matter include living biomass and the release of exudates and other secretions. This is because as microalgae grow and multiply, they form a living biomass that directly constitutes organic matter such as proteins, lipids, and carbohydrates (Renuka et al., 2017). These biomolecules are released into the soil during the decomposition process, contributing to the organic carbon and biopolymer content (Adamu Ugya et al., 2023). Microalgae also actively release a significant portion of the photosynthates they produce as exudates and secretions such as EPS, which enhances soil aggregation and promotes microbial activity (Ugya et al., 2025a). These exudates contain complex organic compounds that serve as a food source for soil microorganisms, further enriching the soil organic matter content (Zhang et al., 2025b). The release of microalgae biomass into the soil also creates a positive feedback loop that further boosts organic content (Ma et al., 2024). This is because the microalgae exudates produced by microalgae are rich carbon sources for heterotrophic bacteria and fungi. This priming effect accelerates the decomposition of organic matter in the soil, leading to increased nutrient availability for plants (Riekenberg et al., 2020). The influx of food caused by the presence of microalgae biomass causes the multiplication of decomposer populations. Microbial multiplication also triggers the release of necromass from decomposers that consume microalgae-derived carbon. This process ultimately enhances soil fertility and promotes plant growth by cycling nutrients more efficiently. Also, the increased microbial activity can improve soil structure and water retention, further benefiting plant health (Ren et al., 2025).

## 3 Mechanism of microalgae and cyanobacteria resources as a biostimulant and biopesticides

The ability of microalgae and cyanobacteria to act as biostimulants and biopesticides involves a complex mechanism (Parmar et al., 2023). This complex mechanism is linked to their rich biochemical composition, which directly influences plant physiology and soil health. For example, microalgae produce metabolites such as vitamins, amino acids, phytohormones, betaines, and alginate-derived oligosaccharides, which can act as biostimulants by improving seed germination, root development, chlorophyll synthesis, and overall plant growth (Zhang et al., 2024a). They also increase stress tolerance in plants by enhancing antioxidant activity and promoting nutrient uptake (Brito-Lopez et al., 2025). Microalgae also produce antimicrobial compounds that can help protect plants from pathogens and diseases. These antimicrobial compounds are able to assist plants to suppress fungal pathogens such as *Fusarium oxysporum* and *Rhizoct onia solani*. For example, Schmid et al. (2022) reported the ability of the extract of *Scenedesmus obliquus* to inhibit the growth of *Sclerotium rolfsii* by 32%. While the extract of *Phaeodactylum tricornutum* suppressed the growth of *Rhizoctonia solani* by 18.35%. This result indicates the prospect of microalgae biopesticide in the reduction of the synthetic pesticides utilization in agriculture, which can help minimize the environmental impact of chemical pesticides (Schmid et al., 2022). The mechanism of microalgae in the enhancement of nutrient uptake and assimilation in plants is primarily through the release of organic compounds that stimulate root growth and increase nutrient availability in the soil. For example, microalgae secrete organic acids such as citric and gluconic acids, which chelate nutrients in the soil, making them more accessible to plants. They also produce phytohormones such as auxins, cytokinins, and gibberellins that stimulate root branching and lengthening, further enhancing the plant’s ability to absorb nutrients (Prisa and Spagnuolo, 2023).

Also, extracts produced from microalgae contain auxins, cytokinins, gibberellins, brassinosteroids, and jasmonates that can promote plant growth, increase resistance to stress, and improve overall health. These bioactive compounds have shown promising results in agricultural applications, offering a sustainable and eco-friendly alternative to traditional synthetic plant growth regulators, as shown in **Table 3** (Mazzafera, 2025). For example, studies have demonstrated that microalgae extracts can enhance seed germination rates, root development, and nutrient uptake in various crops. These extracts have been found to stimulate the production of plant hormones and enzymes involved in stress response mechanisms, leading to improved crop productivity and resilience. For example, microalgae synthesize antioxidants such as carotenoids, phenolics, vitamin E, and glutathione that can help plants combat oxidative stress and increase their tolerance to environmental challenges such as drought, salinity, UV, and temperature (Prisa et al., 2024). Microalgae also have the capacity to supply compatible solutes such as proline and polysaccharides that can help plants maintain cellular osmotic balance and survive under extreme conditions (Ren et al., 2025). They also have the potential to trigger Systemic Acquired Resistance (SAR) in plants, thus activating defense-related enzymes such as peroxidase, polyphenol oxidase, chitinase, and β-1,3-glucanase in plants. This can enhance the plant’s immune response and overall resilience to biotic stressors such as pathogens and pests (Yu et al., 2022).

**Table 3:** Biostimulant produce by microalgae involved in enhancing plant growth.

Microalgae species	Mechanism and key effect on plant growth	Reference:
*Nostoc* sp	Releases indole-3-acetic acid (up to 21 pmol mg/Chl a) that promotes root colonization and enhances rice and wheat growth under axenic conditions.	(Hussain et al., 2015)
*Chlorella vulgaris*	Mitigates cadmium toxicity in rice by absorbing Cd and reducing oxidative stress, thereby improving growth and photosynthetic activity.	(Yotsova et al., 2022)
*Arthrospira platensis*	Microalgae-derived biochar (0.5 mg/mL) increases rice seed yield (44%) and seed weight (53%) by reallocating proteins toward seed development.	(Minello et al., 2024)
*Chlorococcum* sp *Micractinium* sp *Scenedesmus* sp *Chlorella* sp	Extracts enhance spinach germination and biomass up to twofold via production of cytokinins, gibberellins, and auxins, offering a natural alternative to synthetic stimulants.	(Rupawalla et al., 2022)
Mixed microalgal consortia	Extracts (40–60%) used for seed priming and foliar spray improve tomato growth, increasing shoot and root lengths by > 40%.	(Supraja et al., 2020)
*Spirulina* sp	Extract (0.25–0.5%) elevates antioxidant content (74%) and enhances peroxidase and ascorbate peroxidase activities in Cape gooseberry.	(Heydarnajad Giglou et al., 2024)
*Chlorella vulgaris*, *Nannochloropsis salina*, *Arthrospira platensis*	Extracts (0.5%) enhance bean growth and nutritional quality by reducing oxidative stress; *N. salina* shows highest gibberellin (74.9 mg/100 g) and auxin levels.	(Gharib et al., 2024)
*Spirulina* sp	Combined microalgal and macroalgal (*Padina pavonica*) applications increase sesame antioxidant levels, mineral content, and yield traits.	(El-Shazoly et al., 2025)
*Nannochloropsis gaditana* *Porphyridium* sp.	Foliar extracts boost lettuce biomass (+31%), leaf number, and height by enhancing nitrogen assimilation and water-use efficiency.	(Di Serio et al., 2025)
*Chlamydomonas* sp. *Chlorella* sp. *Desmodesmus* sp.	Extracts promote rice growth and yield via exopolysaccharide and siderophore production; root and shoot growth increase by 43% and 36%.	(Lamb et al., 2023)

The mechanisms of microalgae biopesticides are based on the production of bioactive compounds that can disrupt the growth and development of pests. These compounds can act as repellents, toxins, or growth inhibitors, providing an eco-friendly alternative to synthetic pesticides. The metabolites produced by microalgae include secondary metabolites such as phenolics, terpenoids, alkaloids, fatty acids, and peptides (Chaïb et al., 2021). These compounds exhibit antibacterial, antifungal, and antiviral activity because they cause the disruption of pathogen cell membranes, inhibit enzymatic processes, or interfere with cellular communication by blocking bacteria quorum sensing. This enhances plant yield because they help protect plants from harmful pathogens and pests, allowing them to grow more efficiently and produce higher yields. Certain microalgae are able to produce allelochemicals that can also suppress weed growth and competition, further benefiting plant growth. These allelochemicals can help improve soil health and reduce the need for synthetic herbicides, making microalgae a sustainable option for enhancing agricultural productivity (Bhardwaj et al., 2025). Microalgae also produce fatty acids and pigments such as astaxanthin and β-carotene, which have insecticidal and nematicidal effects. These compounds can help reduce the reliance on chemical pesticides in agriculture, promoting a more environmentally friendly approach to pest management. Also, microalgae can be easily integrated into existing farming practices, making them a convenient and cost-effective solution for improving crop yields (Casanova et al., 2023).

## 4 Sustainable utilization of microalgae and cyanobacteria resources as biofertilizers and biostimulants

Microalgae are increasingly recognized as sustainable alternatives to chemical fertilizers and synthetic plant growth regulators due to their ecological benefits, nutrient content, and potential for circular bioeconomy integration. The waste-to-resource model of microalgae increases its utilization for biofertilizer because it allows for the conversion of organic waste into valuable nutrients for plants. For example, microalgae can utilize nutrients from agricultural runoff, municipal wastewater, or industrial effluents for biomass production, which can then be used as a nutrient-rich biofertilizer for crops. This not only helps in reducing waste and pollution but also promotes sustainable agriculture practices by closing the nutrient loop in a circular economy. The cultivating of *Chlorella* sp. in wastewater recovery has led to the recovery of more than 90% of nitrogen and 80% of phosphorus. These recovered nutrients are then slowly released back into the soil, providing a continuous source of nourishment for plants (Ortega-Blas et al., 2025). Also, the ability of microalgae to sequester carbon makes them a promising solution for reducing greenhouse gas emissions. They are able to fix atmospheric CO_2_ through photosynthesis at approximately 1.8 tons of CO_2_ per ton of algal biomass produced. This allows for integration of microalgae cultivation with industrial emissions, thus reducing the cost of microalgae biofertilizer production. This increases the sustainability of both the agricultural and industrial sectors because it aligns with the concept of a circular economy (Geng et al., 2025).

The high nutrient density per input of microalgae biomass also contributes to its potential as a sustainable biofertilizer for enhancing crop productivity. This is attributed to the fact that microalgal biomass is highly concentrated with essential nutrients and bioactive compounds compared to traditional compost or manure. Also, the rapid growth rate of microalgae allows for continuous production of biomass, making it a reliable source of biofertilizer. This can help reduce the reliance on chemical fertilizers, promoting environmentally friendly agricultural practices (Atzori et al., 2020). Microalgae biofertilizer reduced the dependence on nonrenewable inputs, which are associated with mined phosphate or Haber–Bosch nitrogen, leading to a shift towards agricultural sustainability (Slocombe et al., 2020). This transition addresses the severe environmental and economic costs embedded in conventional fertilizers (Ugya et al., 2021). This is because chemical fertilizers depend on phosphate and nitrogen mined from phosphate rock and Haber-Bosch nitrogen (Asadu et al., 2024). The Haber-Bosch process for nitrogen fixation requires high energy inputs, contributing to greenhouse gas emissions and environmental degradation (Ladha et al., 2022). Similarly, phosphate rock mining is unsustainable and leads to habitat destruction and water pollution (Amann et al., 2018). Also, the application of chemical fertilizers can lead to soil degradation and nutrient runoff, further exacerbating environmental issues (Nuruzzaman et al., 2025). Microalgae biofertilizer offers a sustainable alternative by utilizing nutrient-rich algae to provide essential nutrients for plant growth. This method not only reduces reliance on harmful chemical fertilizers but also helps to improve soil health and minimize environmental impact (Hoque et al., 2025).

## 5 Validation challenges limiting scale-up application

The scale-up of microalgae biofertilizer is hindered by a complex interconnected set of challenges ranging from production economics, product efficacy, and regulatory frameworks (Zhang et al., 2025a). The production economics basically involve the costs of cultivation, harvesting, processing, and distribution, which critically shape how widely and effectively microalgae-based biofertilizers can be adopted. This has limited the adoption of microalgae biofertilizer because for maximum biomass production, microalgae cultivation requires controlled environments with adequate light, nutrients, carbon dioxide, and water. Thus, depending on the system used, the capital and operational costs can vary significantly, making it challenging for producers to achieve cost competitiveness with traditional fertilizers. For example, open pond systems are cheaper to build but yield lower productivity due to contamination and environmental fluctuations (Skifa et al., 2025). But closed photobioreactor systems offer higher productivity but come with higher initial investment costs. Also, the cost of the nutrient inputs can greatly impact the overall expenses of microalgae cultivation, as high-quality nutrients can be expensive. Therefore, finding a balance between cost and productivity is crucial for successful microalgae production (Magalhães et al., 2022). Another issue affecting production economics is harvesting and downstream processing, due to labor and energy intensity. This is because techniques such as centrifugation, filtration, or flocculation are required. Although for biofertilizer purposes cheaper methods such as gravity sedimentation or bioflocculation can be used because lower purity is needed compared to other applications like pharmaceuticals. However, optimizing these processes to maintain nutrient content and biological activity while reducing energy input is key to economic viability (Anthony et al., 2013).

The efficacy of a microalgae-based biofertilizer is another limitation to scale-up production. For example, microalgae are known for their rich biochemical composition, including proteins, amino acids, phytohormones, and micronutrients (Miranda et al., 2024). However, the real-world application of this biofertilizer may not always result in the expected crop yield increase due to various factors such as environmental conditions, soil type, and application method (Cao et al., 2023). Also, microalgal performance varies depending on species selection, cultivation conditions, and formulation stability. For example, environmental factors such as pH, light availability, and soil microbiota interactions can affect the bioavailability of algal nutrients and metabolites. Therefore, a lot of questions remain regarding the optimization of microalgal biofertilizers for different agricultural settings and crops (Ugya et al., 2025b).

The regulatory framework also influences the development and application of microalgae as biofertilizers. This is attributed to the need to determine the classification, safety standards, and market approval processes of microalgae-based fertilizer. The high regulation on biofertilizer imposed by many countries creates a barrier for the scale-up production of microalgae-based biofertilizer. Also, the complex regulatory requirements may also impact the cost-effectiveness and commercial viability of microalgae biofertilizers (Santos et al., 2024). To counteract these limitations, the following strategies can be implemented to improve the overall effectiveness of microlagae-based biofertilizer.

- The shift from a standalone microalgae cultivation process to integrated systems. This will provide the platform that links low-cost cultivation with energy-efficient harvesting. For example, the use of waste-derived media such as wastewater or agricultural runoff can reduce production costs and increase sustainability.
- The incorporation of genetic engineering techniques to enhance nutrient uptake and biomass productivity can further improve the efficiency and effectiveness of biofertilizers.
- To eradicate barriers related to product inconsistency and performance. Future work should prioritize the creation of standardized protocols for the cultivation, harvesting, and formulation of biofertilizer products. This can be achieved by establishing clear, data-driven application guidelines that ensure quality control and reproducibility across different production sites. This is because for microalgae biofertilizers to gain regulatory and market acceptance, comprehensive techno-economic analysis (TEA) and life cycle assessment (LCA) are crucial to quantitatively proving the economic viability and net environmental benefits of using these products compared to traditional fertilizers.
- It is crucial for stakeholders to work together to streamline regulations and establish clear guidelines for the production and use of microalgae biofertilizers.

## 6 Conclusion

Microalgae and cyanobacteria act through several mechanisms as biofertilizers by fixing atmospheric nitrogen, solubilizing phosphorus, and releasing essential nutrients. This has the potential to reduce the reliance on chemical fertilizers, decrease environmental pollution, and improve soil health. Furthermore, the use of microalgae and cyanobacteria as biofertilizers can also enhance crop productivity and increase resilience to environmental stressors. They also act as biostimulants through the secretion of plant growth hormones like gibberellins that enhance seed germination, root development, and crop biomass. This biostimulant potential has led to increased interest in utilizing microalgae and cyanobacteria in sustainable agriculture practices as a natural alternative to synthetic growth promoters. Their role as biopesticides involves enriching the soil with beneficial microorganisms that control plant pathogens, and they can also directly contribute to the biodegradation of environmental pesticide residues. These biopesticides are considered environmentally friendly alternatives to chemical pesticides, as they do not harm beneficial insects or contaminate water sources. Despite the benefits of microalgae-based biofertilizer, large-scale application is limited by production economics, product efficacy, and regulatory frameworks. Thus, warranting further research and development to address these challenges and maximize the potential of microalgae biofertilizers in sustainable agriculture practices.

## References

[B1] Adamu Ugya Y. Chen H. Sheng Y. Ajibade F. O. Wang Q. (2023). A review of microalgae biofilm as an eco-friendly approach to bioplastics, promoting environmental sustainability. Environ. Res. 236, 116833. doi: 10.1016/j.envres.2023.116833, PMID: 37543134

[B2] Afkairin A. Ippolito J. A. Stromberger M. Davis J. G. (2021). Solubilization of organic phosphorus sources by cyanobacteria and a commercially available bacterial consortium. Appl. Soil Ecol. 162, 103900. doi: 10.1016/j.apsoil.2021.103900

[B3] Ahmad M. F. Ahmad F. A. Alsayegh A. A. Zeyaullah M. Alshahrani A. M. Muzzammil K. . (2024). Pesticides impacts on human health and the environment with their mechanisms of action and possible countermeasures. Heliyon 10, e29128. doi: 10.1016/j.heliyon.2024.e29128, PMID: 38623208 PMC11016626

[B4] Akca M. O. Saygin S. D. Bilgin A. Arslan S. Erpul G. (2025). Harnessing Chlorella vulgaris for mucilage mitigation and wheat growth on Fluvisol: A study on wastewater treatment efficacy. J. Soil Sci. Plant Nutr. 25, 6720–6739. doi: 10.1007/s42729-025-02559-w

[B5] Al-Shammary A. A. G. Al-Shihmani L. S. S. Fernández-Gálvez J. Caballero-Calvo A. (2024). Optimizing sustainable agriculture: A comprehensive review of agronomic practices and their impacts on soil attributes. J. Environ. Manage. 364, 121487. doi: 10.1016/j.jenvman.2024.121487, PMID: 38889650

[B6] Alvarez A. L. Weyers S. L. Gardner R. D. (2024). Cyanobacteria-based soil amendments in the soil-plant system: Effects of inoculations on soil nutrient and microbial dynamics under spring wheat growth. Algal Res. 77, 103326. doi: 10.1016/j.algal.2023.103326

[B7] Alvarez A. L. Weyers S. L. Goemann H. M. Peyton B. M. Gardner R. D. (2021). Microalgae, soil and plants: A critical review of microalgae as renewable resources for agriculture. Algal Res. 54, 102200. doi: 10.1016/j.algal.2021.102200

[B8] Amann A. Zoboli O. Krampe J. Rechberger H. Zessner M. Egle L. (2018). Environmental impacts of phosphorus recovery from municipal wastewater. Resources Conserv. Recycling 130, 127–139. doi: 10.1016/j.resconrec.2017.11.002

[B9] Anthony R. J. Ellis J. T. Sathish A. Rahman A. Miller C. D. Sims R. C. (2013). Effect of coagulant/flocculants on bioproducts from microalgae. Bioresource Technol. 149, 65–70. doi: 10.1016/j.biortech.2013.09.028, PMID: 24084206

[B10] Asadu C. O. Ezema C. A. Ekwueme B. N. Onoh C. E. Onoh I. M. Adejoh T. . (2024). Enhanced efficiency fertilizers: Overview of production methods, materials used, nutrients release mechanisms, benefits and considerations. Environ. pollut. Manage. 1, 32–48. doi: 10.1016/j.epm.2024.07.002

[B11] Atzori G. Nissim W. G. Rodolfi L. Niccolai A. Biondi N. Mancuso S. . (2020). Algae and bioguano as promising source of organic fertilizers. J. Appl. Phycology 32, 3971–3981. doi: 10.1007/s10811-020-02261-7

[B12] Barahoei M. Kasiri R. Hosseini-Nezhad S. A. Hatamipour M. S. (2024). Production of iron-rich biomass using Chlorella vulgaris cultivation under iron stress. Algal Res. 78, 103395. doi: 10.1016/j.algal.2024.103395

[B13] Bhardwaj R. Yadav A. Sahoo A. Kumari P. Singh L. A. Swapnil P. . (2025). Microalgal-based sustainable bio-fungicides: A promising solution to enhance crop yield. Discover Sustainability 6, 39. doi: 10.1007/s43621-025-00795-9

[B14] Black K. Osborne B. (2004). An assessment of photosynthetic downregulation in cyanobacteria from the Gunnera–Nostoc symbiosis. New Phytol. 162, 125–132. doi: 10.1111/j.1469-8137.2004.01008.x

[B15] Bossa R. Di Colandrea M. Salbitani G. Carfagna S. (2024). Phosphorous utilization in microalgae: Physiological aspects and applied implications. Plants 13, 2127. doi: 10.3390/plants13152127, PMID: 39124245 PMC11314164

[B16] Boutahiri S. Benrkia R. Tembeni B. Idowu O. E. Olatunji O. J. (2024). Effect of biostimulants on the chemical profile of food crops under normal and abiotic stress conditions. Curr. Plant Biol. 40, 100410. doi: 10.1016/j.cpb.2024.100410

[B17] Brito-Lopez C. van der Wielen N. Barbosa M. Karlova R. (2025). Plant growth-promoting microbes and microalgae-based biostimulants: Sustainable strategy for agriculture and abiotic stress resilience. Philos. Trans. R. Soc. B: Biol. Sci. 380, 20240251. doi: 10.1098/rstb.2024.0251, PMID: 40439314 PMC12132076

[B18] Cao T. N.-D. Mukhtar H. Le L.-T. Tran D. P.-H. Ngo M. T. T. Pham M.-D.-T. . (2023). Roles of microalgae-based biofertilizer in sustainability of green agriculture and food-water-energy security nexus. Sci. Total Environ. 870, 161927. doi: 10.1016/j.scitotenv.2023.161927, PMID: 36736400

[B19] Casanova L. M. Macrae A. de Souza J. E. Neves Junior A. Vermelho A. B. (2023). The potential of allelochemicals from microalgae for biopesticides. Plants 12, 1896. doi: 10.3390/plants12091896, PMID: 37176954 PMC10181251

[B20] Chaïb S. Pistevos J. C. A. Bertrand C. Bonnard I. (2021). Allelopathy and allelochemicals from microalgae: An innovative source for bio-herbicidal compounds and biocontrol research. Algal Res. 54, 102213. doi: 10.1016/j.algal.2021.102213

[B21] Colla G. Hoagland L. Ruzzi M. Cardarelli M. Bonini P. Canaguier R. . (2017). Biostimulant action of protein hydrolysates: Unraveling their effects on plant physiology and microbiome. Front. Plant Sci. 8, 2202. doi: 10.3389/fpls.2017.02202, PMID: 29312427 PMC5744479

[B22] Costa O. Y. A. Raaijmakers J. M. Kuramae E. E. (2018). Microbial extracellular polymeric substances: Ecological function and impact on soil aggregation. Front. Microbiol. 9, 1636. doi: 10.3389/fmicb.2018.01636, PMID: 30083145 PMC6064872

[B23] De Silva A. G. S. D. Hashim Z. K. Solomon W. Zhao J.-B. Kovács G. Kulmány I. M. . (2024). Unveiling the role of edaphic microalgae in soil carbon sequestration: Potential for agricultural inoculants in climate change mitigation. Agriculture 14, 2065. doi: 10.3390/agriculture14112065

[B24] Di Serio A. Alfano V. Tava A. Biazzi E. Cappetta E. Del Regno C. . (2025). Marine microalgae extracts as plant biostimulant to boost baby leaf lettuce production. Sci. Rep. 15, 32825. doi: 10.1038/s41598-025-18104-9, PMID: 40999012 PMC12464166

[B25] Do Nascimento M. Battaglia M. E. Sanchez Rizza L. Ambrosio R. Arruebarrena di Palma A. Curatti L. (2019). Prospects of using biomass of N_2_-fixing cyanobacteria as an organic fertilizer and soil conditioner. Algal Res. 43, 101652. doi: 10.1016/j.algal.2019.101652

[B26] El-Shazoly R. M. Yousef S. Hifney A. F. Abdel-Wahab D. A. (2025). Effectiveness of algae as a low-cost alternative input to stimulate Sesamum indicum L. growth and productivity for sustainable purposes. J. Soil Sci. Plant Nutr. 25, 8006–8025. doi: 10.1007/s42729-025-02651-1

[B27] Faruque M. O. Uddin S. Hossain M. M. Hossain S. M. Z. Shafiquzzaman M. Razzak S. A. (2024). A comprehensive review on microalgae-driven heavy metals removal from industrial wastewater using living and nonliving microalgae. J. Hazardous Materials Adv. 16, 100492. doi: 10.1016/j.hazadv.2024.100492

[B28] Ferreyra-Suarez D. García-Depraect O. Castro-Muñoz R. (2024). A review on fungal-based biopesticides and biofertilizers production. Ecotoxicology Environ. Saf. 283, 116945. doi: 10.1016/j.ecoenv.2024.116945, PMID: 39222612

[B29] Gamage A. Gangahagedara R. Subasinghe S. Gamage J. Guruge C. Senaratne S. . (2024). Advancing sustainability: The impact of emerging technologies in agriculture. Curr. Plant Biol. 40, 100420. doi: 10.1016/j.cpb.2024.100420

[B30] Geng Y. Shaukat A. Azhar W. Raza Q-U-A. Tahir A. Abideen M. Z. U. . (2025). Microalgal biorefineries: A systematic review of technological trade-offs and innovation pathways. Biotechnol. Biofuels Bioproducts 18, 93. doi: 10.1186/s13068-025-02694-7, PMID: 40817213 PMC12357411

[B31] Gharib F. A. E. L. Osama K. Sattar A. M. A. E. Ahmed E. Z. (2024). Impact of Chlorella vulgaris, Nannochloropsis salina and Arthrospira platensis as biostimulants on common bean plant growth, yield and antioxidant capacity. Sci. Rep. 14, 1398. doi: 10.1038/s41598-023-50040-4, PMID: 38228623 PMC10791689

[B32] Gheda S. F. Ahmed D. A. (2015). Improved soil characteristics and wheat germination as influenced by inoculation of Nostoc kihlmani and Anabaena cylindrica. Rendiconti Lincei 26, 121–131. doi: 10.1007/s12210-014-0351-8

[B33] Gholami M. Alikhani H. A. Etesami H. Noroozi M. Inglett P. (2025). Enhancing biological nitrogen fixation in rice (Oryza sativa L.) cultivation through diazotrophs-enriched periphyton biofilm. J. Plant Growth Regul. doi: 10.1007/s00344-025-11904-3

[B34] Ghorbani E. Nowruzi B. Nezhadali M. Hekmat A. (2022). Metal removal capability of two cyanobacterial species in autotrophic and mixotrophic mode of nutrition. BMC Microbiol. 22, 58. doi: 10.1186/s12866-022-02471-8, PMID: 35176992 PMC8851847

[B35] Giner-Lamia J. Pereira S. B. Bovea-Marco M. Futschik M. E. Tamagnini P. Oliveira P. (2016). Extracellular proteins: Novel key components of metal resistance in cyanobacteria? Front. Microbiol. 7, 878. doi: 10.3389/fmicb.2016.00878, PMID: 27375598 PMC4894872

[B36] Gonçalves J. Freitas J. Fernandes I. Silva P. (2023). Microalgae as biofertilizers: A sustainable way to improve soil fertility and plant growth. Sustainability 15, 12413. doi: 10.3390/su151612413

[B37] Govindasamy P. Muthusamy S. K. Bagavathiannan M. Mowrer J. Jagannadham P. T. K. Maity A. . (2023). Nitrogen use efficiency—a key to enhance crop productivity under a changing climate. Front. Plant Sci. 14, 1121073. doi: 10.3389/fpls.2023.1121073, PMID: 37143873 PMC10151540

[B38] Heydarnajad Giglou R. Torabi Giglou M. Hatami M. Ghorbanpour M. (2024). Potential of natural stimulants and spirulina algae extracts on Cape gooseberry plant: A study on functional properties and enzymatic activity. Food Sci. Nutr. 12, 9056–9068. doi: 10.1002/fsn3.4342, PMID: 39619967 PMC11606845

[B39] Hoque M. M. Iannelli V. Padula F. Radice R. P. Saha B. K. Martelli G. . (2025). Microalgae: Green engines for achieving carbon sequestration, circular economy, and environmental sustainability – a review based on last ten years of research. Bioengineering (Basel) 12 (9), 909. doi: 10.3390/bioengineering12090909, PMID: 41007154 PMC12467341

[B40] Hussain A. Shah S. T. Rahman H. Irshad M. Iqbal A. (2015). Effect of IAA on *in vitro* growth and colonization of Nostoc in plant roots. Front. Plant Sci. 6. doi: 10.3389/fpls.2015.00046, PMID: 25699072 PMC4318279

[B41] Jiménez-Ríos L. Torrado A. González-Pimentel J. L. Iniesta-Pallarés M. Molina-Heredia F. P. Mariscal V. . (2024). Emerging nitrogen-fixing cyanobacteria for sustainable cotton cultivation. Sci. Total Environ. 924, 171533. doi: 10.1016/j.scitotenv.2024.171533, PMID: 38458446

[B42] Jurado-Flores A. Heredia-Martínez L. G. Torres-Cortes G. Díaz-Santos E. (2025). Harnessing microalgae and cyanobacteria for sustainable agriculture: Mechanistic insights and applications as biostimulants, biofertilizers and biocontrol agents. Agriculture 15, 1842. doi: 10.3390/agriculture15171842

[B44] Kannaiyan S. Ganesan G. (2002). *Azolla–Anabaena* biological symbiotic system for rice production. Madras Agric. J. 89, 185–197. doi: 10.29321/MAJ.10.A00198

[B45] Khoso M. A. Wagan S. Alam I. Hussain A. Ali Q. Saha S. . (2024). Impact of plant growth-promoting rhizobacteria (PGPR) on plant nutrition and root characteristics: Current perspective. Plant Stress 11, 100341. doi: 10.1016/j.stress.2023.100341

[B46] Kollmen J. Juvigny-Khenafou N. P. D. Wastian K. Yavuz S. Engl A. Strieth D. (2025). Co-cultivation of wheat and Arabidopsis thaliana with nitrogen-fixing cyanobacteria: A strategy to enhance plant growth and drought tolerance. J. Appl. Phycology. doi: 10.1007/s10811-025-03677-9

[B47] Ladha J. K. Peoples M. B. Reddy P. M. Biswas J. C. Bennett A. Jat M. L. . (2022). Biological nitrogen fixation and prospects for ecological intensification in cereal-based cropping systems. Field Crops Res. 283, 108541. doi: 10.1016/j.fcr.2022.108541, PMID: 35782167 PMC9133800

[B48] Laje K. Seger M. Dungan B. Cooke P. Polle J. Holguin F. O. (2019). Phytoene accumulation in the novel microalga Chlorococcum sp. using the pigment synthesis inhibitor fluridone. Mar. Drugs 17 (3), 187. doi: 10.3390/md17030187, PMID: 30909380 PMC6471924

[B49] Lamb T. I. Berghahn E. Pita F. M. de Oliveira Neves L. dos Reis Blasi É.A. Hofstetter J. S. . (2023). Isolation and selection of microalgae capable of stimulating rice plant development and seed production. Algal Res. 74, 103203. doi: 10.1016/j.algal.2023.103203

[B50] Li S. Huang W. Peng C. Jing X. Ding J. Chen T. . (2025). Enhancement of rice production and soil carbon sequestration utilizing nitrogen-fixing cyanobacteria. Appl. Soil Ecol. 207, 105940. doi: 10.1016/j.apsoil.2025.105940

[B51] Luo Y. Lu X. Zhou G. Shen H. Li H. Li S. . (2025). Microalgae for phosphorus chemical wastewater treatment and recovery of phosphorus. Environ. Res. 276, 121511. doi: 10.1016/j.envres.2025.121511, PMID: 40174743

[B53] Ma X. Huang D. Huang C. Tong Y. Yuan F. Ma X. . (2023). The application of nitrogen, phosphorus, and potassium regulate the growth and morphological development of Torreya grandis (Taxaceae) saplings. Horticulturae 9, 1203. doi: 10.3390/horticulturae9111203

[B52] Ma F. Li Y. Han X. Li K. Zhao M. Guo L. . (2024). Microalgae-based biofertilizer improves fruit yield and controls greenhouse gas emissions in a hawthorn orchard. PloS One 19, e0307774. doi: 10.1371/journal.pone.0307774, PMID: 39093909 PMC11296634

[B54] Magalhães I. B. Ferreira J. Castro J. D. S. Assis L. R. D. Calijuri M. L. (2022). Agro-industrial wastewater-grown microalgae: A techno-environmental assessment of open and closed systems. Sci. Total Environ. 834, 155282. doi: 10.1016/j.scitotenv.2022.155282, PMID: 35447175

[B55] Mahmud A. A. Upadhyay S. K. Srivastava A. K. Bhojiya A. A. (2021). Biofertilizers: A nexus between soil fertility and crop productivity under abiotic stress. Curr. Res. Environ. Sustainability 3, 100063. doi: 10.1016/j.crsust.2021.100063

[B56] Maqubela M. P. Mnkeni P. N. S. Issa O. M. Pardo M. T. D’Acqui L. P. (2009). Nostoc cyanobacterial inoculation in South African agricultural soils enhances soil structure, fertility, and maize growth. Plant Soil 315, 79–92. doi: 10.1007/s11104-008-9734-x

[B57] Marks E. A. N. Montero O. Rad C. (2019). The biostimulating effects of viable microalgal cells applied to a calcareous soil: Increases in bacterial biomass, phosphorus scavenging, and precipitation of carbonates. Sci. Total Environ. 692, 784–790. doi: 10.1016/j.scitotenv.2019.07.289, PMID: 31539985

[B58] Mazzafera P. (2025). “ Microalgae as biostimulant of plant growth and yield,” in Microalgae and One Health. Eds. Pérez-Gálvez A. Jacob-Lopes E. Queiroz Zepka L. Roca M. (Cambridge, MA: Academic Press), 571–577.

[B59] Minello L. V. P. Kuntzler S. G. Lamb T. I. Neves C. D. O. Berghahn E. da Paschoa R. P. . (2024). Rice plants treated with biochar derived from Spirulina (Arthrospira platensis) optimize resource allocation towards seed production. Front. Plant Sci. 15. doi: 10.3389/fpls.2024.1422935, PMID: 39359626 PMC11444984

[B60] Miranda A. M. Hernandez-Tenorio F. Villalta F. Vargas G. J. Sáez A. A. (2024). Advances in the development of biofertilizers and biostimulants from microalgae. Biology 13, 199. doi: 10.3390/biology13030199, PMID: 38534468 PMC10968465

[B61] Mishra R. Koli D. Sharma V. K. Pabbi S. (2019). Evaluation of growth, nitrogen fixation and P-solubilizing ability of diazotrophic cyanobacteria under mineral phosphorus sources. Indian J. Agric. Sci. 89, 420–425. doi: 10.56093/ijas.v89i3.87581

[B62] Moreno J. Vargas M. A. Olivares H. Rivas J. N. Guerrero M. G. (1998). Exopolysaccharide production by the cyanobacterium Anabaena sp. ATCC 33047 in batch and continuous culture. J. Biotechnol. 60, 175–182. doi: 10.1016/S0168-1656(98)00003-0

[B63] Nawaz T. Fahad S. Gu L. Saud S. Zhou R. (2024a). Cyanobacteria: Role in sustainable biomanufacturing and nitrogen fixation. Biofuels Bioproducts Biorefining 18, 2132–2155. doi: 10.1002/bbb.2674

[B64] Nawaz T. Joshi N. Nelson D. Saud S. Abdelsalam N. R. Abdelhamid M. M. A. . (2024b). Harnessing the potential of nitrogen-fixing cyanobacteria: A rich bio-resource for sustainable soil fertility and enhanced crop productivity. Environ. Technol. Innovation 36, 103886. doi: 10.1016/j.eti.2024.103886

[B65] Nur M. M. A. Mahreni M. Murni S. W. Setyoningrum T. M. Hadi F. Widayati T. W. . (2025). Innovative strategies for utilizing microalgae as dual-purpose biofertilizers and phycoremediators in agroecosystems. Biotechnol. Rep. 45, e00870. doi: 10.1016/j.btre.2024.e00870, PMID: 39758973 PMC11700267

[B66] Nuruzzaman M. Bahar M. M. Naidu R. (2025). Diffuse soil pollution from agriculture: Impacts and remediation. Sci. Total Environ. 962, 178398. doi: 10.1016/j.scitotenv.2025.178398, PMID: 39808904

[B67] Ortega-Blas F. M. Ramos-Saravia J. C. Cossío-Rodríguez P. L. (2025). Removal of nitrogen and phosphorus from municipal wastewater through cultivation of microalgae Chlorella sp. in consortium. Water 17, 1160. doi: 10.3390/w17081160

[B68] Parab S. G. Matondkar S. G. P. (2012). Primary productivity and nitrogen fixation by Trichodesmium spp. in the Arabian Sea. J. Mar. Syst. 105–108, 82–95. doi: 10.1016/j.jmarsys.2012.06.003

[B69] Parmar P. Kumar R. Neha Y. Srivatsan V. (2023). Microalgae as next generation plant growth additives: Functions, applications, challenges and circular bioeconomy-based solutions. Front. Plant Sci. 14. doi: 10.3389/fpls.2023.1073546, PMID: 37063190 PMC10101342

[B70] Prisa D. Fresco R. Jamal A. Saeed M. F. Spagnuolo D. (2024). Exploring the potential of macroalgae for sustainable crop production in agriculture. Life 14, 1263. doi: 10.3390/life14101263, PMID: 39459563 PMC11509091

[B71] Prisa D. Spagnuolo D. (2023). Plant production with microalgal biostimulants. Horticulturae 9, 829. doi: 10.3390/horticulturae9070829

[B72] Rahman A. Fares A. Veettil A. V. Mohtar R. Awal R. (2025). A critical review of the microalgae and cyanobacteria-based biofertilizers: An insight into the cost effectiveness of different algae cultivation strategies. Environ. Technol. Innovation 40, 104480. doi: 10.1016/j.eti.2025.104480

[B73] Ramakrishnan B. Maddela N. R. Venkateswarlu K. Meghara M. (2023). Potential of microalgae and cyanobacteria to improve soil health and agricultural productivity: A critical view. Environ. Sci. Adv. 2, 586–611. doi: 10.1039/D2VA00158F

[B74] Ren C. G. Kong C. C. Li S. M. Wang X. J. Yu X. Wang Y. C. . (2025). Symbiotic microalgae and microbes: A new frontier in saline agriculture. Front. Microbiol. 16, 1540274. doi: 10.3389/fmicb.2025.1540274, PMID: 40330728 PMC12052889

[B75] Renuka N. Prasanna R. Sood A. Bansal R. Bidyarani N. Singh R. . (2017). Wastewater grown microalgal biomass as inoculants for improving micronutrient availability in wheat. Rhizosphere 3, 150–159. doi: 10.1016/j.rhisph.2017.04.005

[B76] Riekenberg P. M. Oakes J. M. Eyre B. D. (2020). Shining light on priming in euphotic sediments: Nutrient enrichment stimulates export of stored organic matter. Environ. Sci. Technol. 54, 11165–11172. doi: 10.1021/acs.est.0c01914, PMID: 32786559

[B77] Roy M. Medhekar A. (2025). Transforming smart farming for sustainability through agri-tech innovations: Insights from the Australian agricultural landscape. Farming System 3, 100165. doi: 10.1016/j.farsys.2025.100165

[B78] Rupawalla Z. Shaw L. Ross I. L. Schmidt S. Hankamer B. Wolf J. (2022). Germination screen for microalgae-generated plant growth biostimulants. Algal Res. 66, 102784. doi: 10.1016/j.algal.2022.102784

[B79] Saleem A. Anwar S. Nawaz T. Fahad S. Saud S. ur Rahman T. . (2025). Securing a sustainable future: The climate change threat to agriculture, food security, and sustainable development goals. J. Umm Al-Qura Univ. Appl. Sci. 11, 595–611. doi: 10.1007/s43994-024-00177-3

[B80] Samoraj M. Çalış D. Trzaska K. Mironiuk M. Chojnacka K. (2024). Advancements in algal biorefineries for sustainable agriculture: Biofuels, high-value products, and environmental solutions. Biocatalysis Agric. Biotechnol. 58, 103224. doi: 10.1016/j.bcab.2024.103224

[B81] Santos F. Melkani S. Oliveira-Paiva C. Bini D. Pavuluri K. Gatiboni L. . (2024). Biofertilizer use in the United States: Definition, regulation, and prospects. Appl. Microbiol. Biotechnol. 108, 511. doi: 10.1007/s00253-024-13347-4, PMID: 39531072 PMC11557716

[B82] Satterthwaite D. McGranahan G. Tacoli C. (2010). Urbanization and its implications for food and farming. Philos. Trans. R. Soc. B: Biol. Sci. 365, 2809–2820. doi: 10.1098/rstb.2010.0136, PMID: 20713386 PMC2935117

[B83] Schmid B. Coelho L. Schulze P. S. C. Pereira H. Santos T. Maia I. B. . (2022). Antifungal properties of aqueous microalgal extracts. Bioresource Technol. Rep. 18, 101096. doi: 10.1016/j.biteb.2022.101096

[B84] Serrani D. Cocco S. Cardelli V. D’Ottavio P. Rafael R. B. A. Feniasses D. . (2022). Soil fertility in slash and burn agricultural systems in central Mozambique. J. Environ. Manage. 322, 116031. doi: 10.1016/j.jenvman.2022.116031, PMID: 36055093

[B85] Shedeed Z. A. Gheda S. Elsanadily S. Alharbi K. Osman M. E. H. (2022). Spirulina platensis biofertilization for enhancing growth, photosynthetic capacity and yield of Lupinus luteus. Agriculture 12, 781. doi: 10.3390/agriculture12060781

[B86] Skifa I. Chauchat N. Cocquet P.-H. Guer Y. L. (2025). Microalgae cultivation in raceway ponds: Advances, challenges, and hydrodynamic considerations. EFB Bioeconomy J. 5, 100073. doi: 10.1016/j.bioeco.2024.100073

[B87] Slocombe S. P. Zúñiga-Burgos T. Chu L. Wood N. J. Camargo-Valero M. A. Baker A. (2020). Fixing the broken phosphorus cycle: Wastewater remediation by microalgal polyphosphates. Front. Plant Sci. 11, 982. doi: 10.3389/fpls.2020.00982, PMID: 32695134 PMC7339613

[B88] Solovchenko A. Khozin-Goldberg I. Selyakh I. Semenova L. Ismagulova T. Lukyanov A. . (2019). Phosphorus starvation and luxury uptake in green microalgae revisited. Algal Res. 43, 101651. doi: 10.1016/j.algal.2019.101651

[B89] Solovchenko A. Zaitsev P. Zotov V. (2021). “ Phosphorus biofertilizer from microalgae,” in Biofertilizers. Eds. Rakshit A. Meena V. S. Parihar M. Singh H. B. Singh A. K. (Sawston, Cambridge: Woodhead Publishing), 57–68.

[B90] Song X. Liu J. Feng Y. Zhou C. Li X. Yan X. . (2024). Microalgae-based biofertilizers improve fertility and microbial community structures in the soil of potted tomato. Front. Plant Sci. 15. doi: 10.3389/fpls.2024.1461945, PMID: 39830944 PMC11740598

[B91] Su Y. Ren Y. Wang G. Li J. Zhang H. Yang Y. . (2025). Microalgae and microbial inoculant as partial substitutes for chemical fertilizer enhance Polygala tenuifolia yield and quality by improving soil microorganisms. Front. Plant Sci. 15. doi: 10.3389/fpls.2024.1499966, PMID: 39886683 PMC11779722

[B43] Supraja K.V. Behera B. Balasubramanian P. (2020). Efficacy of microalgal extracts as biostimulants through seed treatment and foliar spray for tomato cultivation. Ind. Crops Products 151, 112453. doi: 10.1016/j.indcrop.2020.112453

[B92] Tal A. (2025). The environmental impacts of overpopulation. Encyclopedia 5, 45. doi: 10.3390/encyclopedia5020045

[B93] Toribio A. J. Suárez-Estrella F. Jurado M. M. López M. J. López-González J. A. Moreno J. (2020). Prospection of cyanobacteria producing bioactive substances and their application as potential phytostimulating agents. Biotechnol. Rep. (Amsterdam) 26, e00449. doi: 10.1016/j.btre.2020.e00449, PMID: 32368511 PMC7184136

[B94] Trevisan S. Francioso O. Quaggiotti S. Nardi S. (2010). Humic substances biological activity at the plant-soil interface: From environmental aspects to molecular factors. Plant Signaling Behav. 5, 635–643. doi: 10.4161/psb.5.6.11211, PMID: 20495384 PMC3001551

[B95] Ugya A. Y. Ajibade F. O. Hua X. (2021). The efficiency of microalgae biofilm in the phycoremediation of water from River Kaduna. J. Environ. Manage. 295, 113109. doi: 10.1016/j.jenvman.2021.113109, PMID: 34216901

[B96] Ugya A. Y. Chen H. Wang Q. (2023). Microalgae biofilm system as an efficient tool for wastewater remediation and potential bioresources for pharmaceutical product production: An overview. Int. J. Phytoremediation 26, 131–142. doi: 10.1080/15226514.2023.2229920, PMID: 37382505

[B97] Ugya A. Y. Chen H. Wang Q. (2025a). Deciphering the mechanism of nano-enhanced docosahexaenoic acid accumulation in microalgae. J. CO_2_ Utilization 100, 103182. doi: 10.1016/j.jcou.2025.103182

[B98] Ugya A. Y. Chen H. Wang Q. (2025b). Enhancing the sustainability of microalgae cultivation through biosensing technology. Materials Today Sustainability 31, 101139. doi: 10.1016/j.mtsust.2025.101139

[B99] Ugya A. Y. Hasan D. U. B. Ari H. A. Sheng Y. Chen H. Wang Q. (2024a). Antibiotic synergistic effect surge bioenergy potential and pathogen resistance of Chlorella variabilis biofilm. Environ. Res. 259, 119521. doi: 10.1016/j.envres.2024.119521, PMID: 38960350

[B100] Ugya A. Y. Li X. Chen H. Wang Q. (2024b). Microalgae stress sensing through oxidative phosphorylation drives bioenergy potential: Deciphering mechanisms and future opportunities. J. Environ. Chem. Eng. 12, 114266. doi: 10.1016/j.jece.2024.114266

[B101] Ugya A. Y. Sheng Y. Chen H. Wang Q. (2024c). Microalgal bioengineering: A futuristic tool for carbon capture. Results Eng. 24, 102990. doi: 10.1016/j.rineng.2024.102990

[B102] Ugya A. Y. Yan C. Ari H. A. Chen H. Wang Q. (2025c). Salinity stress adaptation enhances metabolic flux towards polyhydroxyalkanoate biosynthesis in microalgae biofilm. Chem. Eng. J. 519, 165423. doi: 10.1016/j.cej.2025.165423

[B103] Umamaheswari A. Madhavi D. R. Venkateswarlu K. (1997). Siderophore production in two species of Nostoc as influenced by the toxicity of nitrophenolics. Bull. Environ. Contamination Toxicol. 59, 306–312. doi: 10.1007/s001289900480, PMID: 9211704

[B104] Umbricht J. Filella A. Klett A. Vogts A. Benavides M. Voss M. (2024). Uptake of dissolved inorganic nitrogen and N_2_ fixation by Crocosphaera watsonii under climate change scenarios. Front. Mar. Sci. 11. doi: 10.3389/fmars.2024.1388214

[B105] Yandigeri M. S. Meena K. K. Srinivasan R. Pabbi S. (2011a). Effect of mineral phosphate solubilization on biological nitrogen fixation by diazotrophic cyanobacteria. Indian J. Microbiol. 51, 48–53. doi: 10.1007/s12088-011-0081-x, PMID: 22282628 PMC3209874

[B106] Yandigeri M. S. Yadav A. K. Srinivasan R. Kashyap S. Pabbi S. (2011b). Studies on mineral phosphate solubilization by cyanobacteria Westiellopsis and Anabaena. Microbiology 80, 558–565. doi: 10.1134/S0026261711040229, PMID: 22073557

[B107] Yasmeen A. R. Maharajan T. Rameshkumar R. Sindhamani S. Banumathi B. Prabakaran M. . (2025). Role of seaweeds for improving soil fertility and crop development to address global food insecurity. Crops 5, 29. doi: 10.3390/crops5030029

[B108] Yotsova E. Stefanov M. Rashkov G. Kouzmanova M. Dobrikova A. Apostolova E. (2022). Microalgae improve the photosynthetic performance of rice seedlings (Oryza sativa L.) under physiological conditions and cadmium stress. Phyton - Int. J. Exp. Bot. 91, 1365–1380. doi: 10.32604/phyton.2022.020566

[B109] Yu Y. Gui Y. Li Z. Jiang C. Guo J. Niu D. (2022). Induced systemic resistance for improving plant immunity by beneficial microbes. Plants 11, 386. doi: 10.3390/plants11030386, PMID: 35161366 PMC8839143

[B113] Zhang Y. Ding S. Xue X. Hu X. Rehman O. U. Liu W. . (2025c). Combined application of nitrogen-fixing cyanobacteria enhances rice growth and nutritional quality in saline environments. Algal Res. 89, 104061. doi: 10.1016/j.algal.2025.104061

[B111] Zhang X. Ning Y. Han L. Zhu Y. Li H. (2025b). Microalgae enhance the ameliorative effects of organic materials on soil treated by thermal desorption: Soil properties and nutrient cycling. J. Environ. Manage. 392, 126737. doi: 10.1016/j.jenvman.2025.126737, PMID: 40773941

[B110] Zhang H. Wu Y. Liu D. Feng S. Xuan X. Dong G. . (2025a). Insights into microalgal biotechnology: Current applications, key challenges, and future prospects. J. Environ. Manage. 394, 127263. doi: 10.1016/j.jenvman.2025.127263, PMID: 40961787

[B114] Zhang Z. Xu M. Fan Y. Zhang L. Wang H. (2024b). Using microalgae to reduce the use of conventional fertilizers in hydroponics and soil-based cultivation. Sci. Total Environ. 912, 169424. doi: 10.1016/j.scitotenv.2023.169424, PMID: 38128652

[B112] Zhang X. Yin J. Ma Y. Peng Y. Fenton O. Wang W. . (2024a). Unlocking the potential of biostimulants derived from organic waste and by-product sources: Improving plant growth and tolerance to abiotic stresses in agriculture. Environ. Technol. Innovation 34, 103571. doi: 10.1016/j.eti.2024.103571

